# Higher cerebral oxygen saturation may provide higher urinary output during continuous regional cerebral perfusion

**DOI:** 10.1186/1749-8090-3-58

**Published:** 2008-10-31

**Authors:** Takashi Miyamoto, Kagami Miyaji, Hirotsugu Okamoto, Satoshi Kohira, Takahiro Tomoyasu, Nobuyuki Inoue, Kuniyoshi Ohara

**Affiliations:** 1 Department of Cardiothoracic Surgery, Kitasato University School of Medicine, Kanagawa, Japan; 2 Department of Anesthesiology, Kitasato University School of Medicine, Kanagawa, Japan; 3 Department of Clinical Engineering, Kitasato University School of Medicine, Kanagawa, Japan

## Abstract

**Objective:**

We examined the hypothesis that higher cerebral oxygen saturation (rSO_2_) during RCP is correlated with urinary output.

**Methods:**

Between December 2002 and August 2006, 12 patients aged 3 to 61 days and weighing 2.6 to 3.4 kg underwent aortic arch repair with RCP. Urinary output and rSO_2 _were analyzed retrospectively. Data were assigned to either of 2 groups according to their corresponding rSO_2_: Group A (rSO_2 _≦ 75%) and Group B (rSO_2 _< 75%).

**Results:**

Seven and 5 patients were assigned to Group A and Group B, respectively.

Group A was characterized by mean radial arterial pressure (37.9 ± 9.6 vs 45.8 ± 7.8 mmHg; *P *= 0.14) and femoral arterial pressure (6.7 ± 6.1 vs 20.8 ± 14.6 mmHg; *P *= 0.09) compared to Group B. However, higher urinary output during CPB (1.03 ± 1.18 vs 0.10 ± 0.15 ml·kg^-1^·h^-1^; *P *= 0.03). Furthermore our results indicate that a higher dose of Chlorpromazine was used in Group A (2.9 ± 1.4 vs 1.7 ± 1.0 mg/kg; *P *= 0.03).

**Conclusion:**

Higher cerebral oxygenation may provide higher urinary output due to higher renal blood flow through collateral circulation.

## Introduction

Many authors have previously reported risk factors for aortic arch reconstruction in pediatric cardiac surgery including the Norwood operation for hypoplastic left heart syndrome [1,2]. A potential risk of complications such as neurologic deficits and renal dysfunction is still a controversy. Regional cerebral perfusion (RCP) has been shown to provide cerebral circulatory support during arch reconstruction, minimizing or avoiding deep hypothermic circulatory arrest (DHCA). The brain receives direct perfusion through the right common carotid artery and right vertebral artery. The ability of RCP to provide sub-diaphragmatic somatic circulatory support via collateral vessels (the arterial circle of Willis, internal mammary arteries, intercostal arteries, etc.) has previously been described in detail [3,4].

Near-infrared spectroscopy (NIRS) is used to measure the oxygen saturation of hemoglobin in the blood vessels of the frontal cerebral cortex. This is a new method in which infrared light at 730 and 810 nm wave lengths is used to measure the absorption spectra of oxyhemoglobin and deoxyhemoglobin in the frontal cerebral cortex. Clearly, the chief proposed use of NIRS technology is as a real-time, noninvasive, on-line monitor of the concentration of cerebral HbO_2_, Hb, and oxidized Cytochrome aa3. It may provide an estimate of cerebral blood flow, the cerebral blood volume, and neurological functions [5]. Thus, we propose the hypothesis that higher cerebral oxygen saturation during RCP is correlated with urinary output. Therefore, the purpose of this study is to evaluate the protective potential of RCP via the innominate artery to supply the subdiaphragmatic parts of the body.

## Methods

Between December 2002 and August 2006, 12 patients aged from 3 to 61 days (mean, 8 days) and weighing 2.6 to 3.4 kg (mean, 2.8 kg) underwent aortic arch reconstruction using regional low flow perfusion (RLFP). The patient who died and had pre-operative renal failure were excluded. Six patients required the Norwood procedure with RV-PA conduit, 4 required arch reconstructions with a biventricular repair, and 2 required arch reconstructions with a univentricular repair. All patients underwent RCP without DHCA. Although neonatal artic arch reconstruction may be required in a variety of conditions, the Norwood operation for hypoplastic left heart syndrome (HLHS) with DHCA is most commonly performed. However, this technique has some risks such as neurological injury and renal failure when the DHCA time increases.

A 3.5 mm polytetrafluorethylene (PTFE) graft is anastomosed to the innominate artery and an 8-French aortic cannula (DLP Medtronic, MI) is inserted into the graft. After bicaval venous cannulation, the patient is cooled on cardiopulmonary bypass (140 to 160 ml·kg^-1^·min^-1^) to 25°C nasopharyngeal temperature with the pulmonary arteries snared. The descending aorta is cross clamped at the proximal of the innominate artery with the left carotid and subclavian artery snared. Pump flow is adjusted (50 to 100 ml·kg^-1^·min^-1^) to achieve a mean arterial pressure between 30 and 50 mmHg of the upper body monitored by a right radial arterial line. An aortic cross clamp is emplaced and crystalloid cardioplegia is administrated. Ductal tissue and aortic coarctation is resected. The RCP is used for the aortic arch reconstruction. The distal aortic arch and the descending aorta are anastomosed in an end-to-side fashion. Continuity between the pulmonary trunk and the aortic arch is established by anastomosis of the anterior part of the pulmonary trunk directly to the aortic arch and to the pericardial patch in place for the remaining part of the pulmonary trunk. Atrial septectomy is performed through a small right atriotomy under the total bypass. A PTFE graft is emplaced between the right ventricle and the distal pulmonary artery (RV-PA conduit) for the pulmonary blood source. Chlorpromazin is administrated and the pH-stat blood gas strategy is used for all patients during cardiopulmonary bypass (CPB).

Standard homodynamic monitoring was applied to all patients. Cerebral physiological monitoring included NIRS to measure cerebral oxygen saturation (rSO_2_). Pump flow rate was adjusted to maintain mean right radial arterial pressure between 30 and 50 mmHg during RLFP. We studied the relationship between rSO_2 _and urinary output during RLFP, CPB and for one day postoperatively.

Laboratory values were measured preoperatively and 24 hours postoperatively at the clinical laboratory at the Kitasato University School of Medicine. Outcome follow-up consisted of a medical record review of all available information for: postoperative mortality and neurologic deficits for 30 days. Preoperative cranial ultrasound and electroencephalogram (EEG) examinations were performed and again before discharging the patient from the hospital. The institutional Committee on Human Research at the Kitasato University School of Medicine approved this observational study.

### Statistical analysis

Data are expressed as median and range or mean ± SD unless otherwise specified. Comparisons of means between 2 groups were performed with independent samples of a *t *test, and for univariate comparison of different variables, Mann-Whitney U test was used when appropriate. Statistical analysis was performed with SPSS for Windows 6.01 (SPSS Inc, Chicago, IL). A *P *value of less than 0.05 was considered statistically significant.

## Results

**Figure 1:**
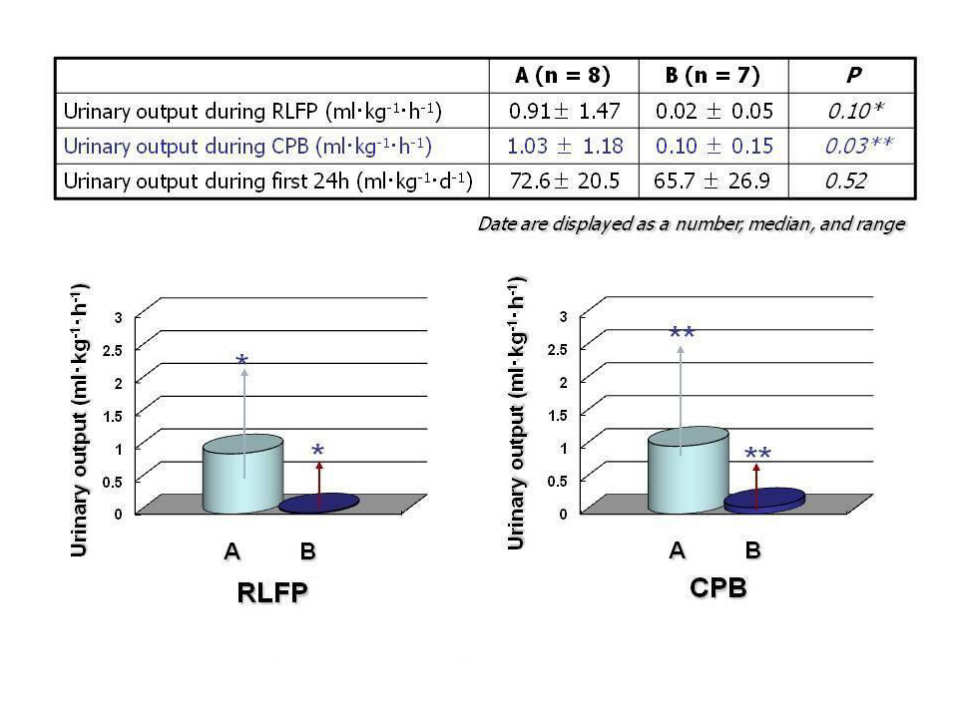
Urinary output during RCP and CPB was significantly higher in Group A than that in Group B.

The mean duration of RCP was 53.0 ± 18.9 min. A mean bypass flow of 141.5 ± 37.0 ml·kg^-1^·min^-1 ^was required to maintain a mean radial arterial pressure of between 30 and 50 mmHg. Cerebral oxygen saturation data differed markedly, however, rSO_2 _was maintained at more than 50% during RCP. Data were assigned to either of 2 groups according to their corresponding rSO_2_. Group A was composed of 7 cases ≦ 75%, and Group B was composed of 5 cases < 75%. Data of the patients' diagnoses and operations are shown in Table 1. Group A was characterized by a similar pump flow rate (79.6 ± 23.0 ml·kg^-1^·min^-1 ^vs 72.7 ± 24.0 ml·kg^-1^·min^-1^; *P *= 0.60) and mean radial arterial pressure (37.9 ± 9.6 mmHg vs 45.8 ± 7.8 mmHg; *P *= 0.14) compared to Group B in Table 2. However, a dose of chlorpromazine (CPZ) (2.9 ± 1.4 mg/kg vs 1.7 ± 1.0 mg/kg; *P *= 0.03) was significantly larger in Group A than in Group B. Finally, urinary output during CPB (1.03 ± 1.18 vs 0.10 ± 0.15 ml·kg^-1^·h^-1^; *P *= 0.03) was significantly higher in Group A than that in Group B (Figure 1).

**Table 1:** Comparison of preoperative and operative variables of the two groups

	Group A (n = 7)	Group B (n = 5)	*P*
Preoperative conditions			
Age (days)	5.6 ± 2.0	33.8 ± 20.7	0.004
Body Weight (kg)	2.8 ± 0.4	3.1 ± 0.4	---
Sex (female/male)	2/5	3/2	---
Norwood procedure	4	2	---
Coarctation repair	3	3	---
Operative factors			
CPB time (min)	145.6 ± 35.3	135.6 ± 42.8	---
ACC time (min)	65.7 ± 29.7	59.0 ± 29.1	---
RCP time (min)	59.6 ± 11.9	43.8 ± 24.2	---

CPB, cardiopulmonary bypass; ACC, aortic cross clamp; RCP, regional cerebral perfusion

**Table 2:** Comparison of operative data during RCP of the two groups

	Group A (n = 8)	Group B (n = 7)	*P*
Pump flow rate (ml/kg/min)	79.6 ± 23.0	72.7 ± 24.0	0.60
Mean right radial arterial pressure (mmHg)	37.9 ± 9.6	45.8 ± 7.8	0.14
Mean femoral arterial pressure (mmHg)	6.7 ± 6.1	20.8 ± 14.6	0.09
Central venous pressure (mmHg)	5.7 ± 2.9	5.4 ± 2.3	0.93
Systemic venous oxygen saturation (%)	97.9 ± 2.3	89.3 ± 10.8	0.07
			
Rectal temperature (°)	26.5 ± 1.3	27.6 ± 2.2	0.39
			
Hemoglobin (mg/dl)	9.5 ± 0.8	8.9 ± 1.1	0.39
pH	7.37 ± 0.11	7.37 ± 0.13	0.72
PCO_2 _(mmHg)	36.7 ± 9.5	35.5 ± 6.8	0.58
Base excess	-4.0 ± 3.0	-6.3 ± 4.6	0.39
Chlorpromazine (mg/kg)	2.9 ± 1.4	1.7 ± 1.0	0.03

Data are shown as mean ± SD.

### Postoperative Course

Pre- and postoperative laboratory values are detailed in Table 3. A significant rise of serum alanine aminotransferase (ALT) and CRP in Group A in comparison to Group B became evident and persisted 24 hours postoperatively. However, there are no significant differences between the two groups one month later postoperatively. Delayed sternal closure was necessary in 5 group A and 5 group B patients. Also, there were no significant differences in the duration of ventilation (6.4 ± 3.1 days vs 5.4 ± 4.5 days, *P *= 0.57), ICU stay (8.2 ± 2.8 days vs 7.8 ± 4.6 days, *P *= 0.99) and hospitalization (39.1 ± 23.7 days vs 38.3 ± 28.6 days, *P *= 0.14) after operation between the two groups.

**Table 3:** Comparison of pre and postoperative laboratory values of the two groups

	Group A (n = 7)	Group B (n = 5)	*P*
AST (IU/l)			
Preoperatve	36.1 ± 11.2	38.8 ± 22.4	0.87
***Postoperative (24 h)***	***48.1 ± 40.7***	***75.0 ± 27.3***	***0.04***
Postoperative (1 month)	35.4 ± 20.0	24.5 ± 4.4	0.45
ALT (IU/l)			
Preoperatve	13.0 ± 3.4	20.0 ± 15.4	0.62
*Postoperative (24 h)*	*10.9 ± 2.3*	*24.2 ± 13.0*	*0.01*
Postoperative (1 month)	26.3 ± 14.9	20.0 ± 2.7	0.78
LDH (IU/l)			
Preoperatve	478.4 ± 81.4	485.4 ± 143.3	0.81
Postoperative (24 h)	549.9 ± 152.2	889.2 ± 372.5	0.08
Postoperative (1 month)	288 ± 143	469 ± 164	0.19
BUN (mg/dl)			
Preoperatve	10.0 ± 6.6	13.2 ± 7.3	0.93
Postoperative (24 h)	21.3 ± 5.8	22.2 ± 8.8	0.68
Postoperative (1 month)	19.0 ± 16.8	11.5 ± 5.7	0.29
Cr (mg/dl)			
Preoperatve	0.59 ± 0.16	0.46 ± 0.22	0.11
Postoperative (24 h)	0.86 ± 0.20	0.74 ± 0.12	0.32
Postoperative (1 month)	0.34 ± 0.13	0.25 ± 0.12	0.39
Hb (g/dl)			
Preoperatve	15.3 ± 2.3	14.4 ± 3.4	0.81
Postoperative (24 h)	14.7 ± 1.5	14.3 ± 1.4	0.63
Postoperative (1 month)	12.8 ± 1.3	13.5 ± 1.8	0.34
PLT (× 10^4^/μl)			
Preoperatve	28.8 ± 8.9	36.0 ± 24.4	0.68
Postoperative (24 h)	22.5 ± 7.3	16.9 ± 7.9	0.29
Postoperative (1 month)	41.7 ± 15.2	40.5 ± 19.5	0.99
CRP (mg/l)			
Preoperatve	232 ± 277	225 ± 333	0.68
***Postoperative (24 h)***	***2303 ± 1357***	***9524 ± 5174***	***0.005***
Postoperative (1 month)	420 ± 918	520 ± 704	0.57

Data are displayed as mean ± SD

AST, aspirate amino-transferase; ALT, alanine amino-transferase; LDH, lactate dehydrogenase; BUN, blood urea nitrogen; Cr, Creatinin; Hb, Hemoglobin; PLT, platelet; CRP, C-reactive protein

All patients underwent or routine preoperative and postoperative cerebral ultrasound studies; even though we have no neurological deficits, other than one case of a deceased patient complicated with cerebral bleeding, there are no neurological deficits in Group A. None of the patients experienced hepatic dysfunction or necrotizing enterocolitis.

## Discussion

There is some evidence that RCP via the innominate artery at 25°C leads to some tissue damage in the musculature of the lower limbs [6]. Antegrade selevtive cerebral perfusion is a safe and effecitive procedure and might improve outcome of neonatal aortic arch surgery, minimizing neurologic impact without the need for deep hypothermia [7]. However, its superior potential in preserving the functional integrity of the brain and spinal cord justifies the expanding application of this perfusion technique. The combination of RCP and pH-stat principles resulted in the best neurological outcome as measured with cortical SSEPs (somatosensory evoked potentials) and resulted in the lower lactate elevation indicating the least tissue damage [6]. In previous reports, the data suggest that the pump flow rate of 20 ml·kg^-1^·min^-1 ^during RCP provides enough to ensure adequate cerebral blood flow in the human neonate [8-10]. RCP can provide blood flow to the brain and maintenance of rSO_2 _has been demonstrated with NIRS [11]. In the present study, rSO_2 _was lower than SvO_2 _(systemic venous oxygen saturation) during RCP. This is why there is a notable limitation of the underestimation of the importance to measure the average saturation of both oxyhemoglobin and deoxyhemoglobin. However, NIRS has aided the study of regional perfusion by providing a noninvasive estimate of rSO_2 _and allow quantification of blood flow requirements of the neonatal brain. In actuality, rSO_2 _varies in response to the perfusion flow rates during RCP and CPB. The relationship between cerebral rSO_2 _and potential determinants (mean radial arterial blood pressure, central venous pressure, SaO_2 _[oxygen percent saturation (arterial)], PaCO_2 _[partial pressure of arterial carbon dioxide], temperature, hematocrit and CPB) is still unclear. Furthermore moderate hypothermia with a mild degree of hemodilution might be effective in protecting other organs from ischemic damage and optimaizing cerebral oxygen supply [7]. In my opinion, the need for increasing cerebral rSO_2 _to reduce neurological morbidity undergoing moderate hypothermia during RCP is keenly felt. Although absolute thresholds of rSO_2 _for neurological injury are not known in human neonates, we think that aerobic metabolism is impaired when cerebral rSO_2 _decreases below 50% and agree with previous investigators [12,13]. We subsequently targeted a cerebral rSO_2 _greater than 75% during moderate hypothermic RCP as a cut point in the present study.

The mean right radial artery blood pressure was 30–50 mmHg with the femoral artery blood pressure of 10 – 25 mmHg during RCP. These findings support the speculations that there is an extensive network of vascular collaterals between the supradiaphragmatic and subdiaphragmatic vasculature in neonates and early infants. These collateral flows are consistent with our observations of urinary output during RCP and CPB. In this study, higher urinary output depends on the higher cerebral oxygen saturation and a large dose of chlorpromazine.

Therefore, we suppose that blood flow is correlated to low resistance and low blood pressure. Decreased cerebral vascular resistance may require higher blood flow volumes. We think that the degree of resistance is easily influenced by hypothermia, blood pH, carbon dioxide tension, venous blood compartment, and medication of Chlorpromazine and α-receptor antagonists that dilate the vasculature [14]. Our results indicate that a higher dose of Chlorpromazine was used in Group A and there were similar doses of milrinone and isoflurane in both groups.

Therefore, we recognize that low resistance in the peripheral artery provides significantly high cerebral blood flow and collateral blood flow during RCP. Higher pump flow rate during RCP may provide higher blood flow to the lower body through collateral circulation, potentially increasing oxygen delivery to the subdiaphragmatic vascular beds and improving organ function [15,16]. The presumed reason for this somatic circulatory support during RCP is the widespread network of arterial collaterals from vessels, such as the arterial circle of Willis, internal thoracic and intercostal arteries that connect to ensure the adequate perfusion of the subdiaphragmatic viscera and lower limbs. From our study we concluded that a reasonable mean left radial artery blood pressure was 30–45 mmHg with the femoral artery blood pressure of 10–15 mmHg during RCP.

Alternatively, anti-diuretic hormone (vasopressin) receptors have been found in aortic smooth muscle, live, brain, anterior pituitary and mesangial cells. It plays a major role in regulating plasma osmolality in the mature subject by its action on water and solute reabsorption in nephrons. I think that its function may have decreased and hydrodiuresis accelerate in neonate than early infant.

Moreover, we have come to these conclusions because there was a significant rise of serum AST, ALT and CRP in Group A compared with Group B. AST and ALT is highly enzyme specific for liver cells and is released in case of their damage. CRP is highly enzyme specific for hypercytoxicemia and is released in case of tissue injury due to hypoperfusion for subdiaphragmatic organs. We recently place the NIRS probes on the Th_10 _– L_2 _posterior flank and somatic oxygen saturation is monitored continuously during CPB. According to the clinical data from 4 patients in this study, the increase in somatic rSO_2 _was related to the increase in cerebral rSO_2 _during RCP. Three patients of Group A depict that the somatic rSO_2 _was greater than 50% during RCP. These findings support an extensive network of vascular collaterals between the supradiaphragmatic and subdiaphragmatic vasculature.

Previous reports describe that circulatory arrest was associated with a higher likelihood of clinical and EEG seizures, a longer time to the recovery of normal brain activities as assessed by EEG, and a greater release of the BB isoenzyme of creatine kinase over the first 6 hours after surgery [17]. Even though definite clinical seizures occurred more frequently among the infants randomly assigned to the circulatory arrest group, there were no neurological deficits other than one patient who suffered from a complication of cerebral bleeding and died. The use of this technique may prevent brain injury, including microembolism, macroembolism, and insufficient cerebral perfusion. Of course, we will verify the neurological outcomes of such events in follow-up studies.

In conclusion, higher cerebral oxygenation may provide higher urinary output due to higher renal blood flow through collateral circulation. These findings support the speculations that there is an extensive network of vascular collaterals between the supradiaphragmatic and subdiaphragmatic vasculature in neonates and early infants. Thus, more than 75% of cerebral oxygenation should be targeted during RLFP controlled by NIRS.

## Competing interests

The authors declare that they have no competing interests.

## Authors' contributions

TM conceived and carried out the clinical studies, participated in the sequence alignment and drafted the manuscript. KM participated in its design and coordination. HO participated in the design of the study and performed the statistical analysis. SK participated in the design of the study and performed the statistical analysis. TT participated in the sequence alignment. NI participated in the sequence alignment. KO participated in its design and coordination. All authors read and approved the final manuscript.
